# Assessing bone mineralisation in children with chronic kidney disease: what clinical and research tools are available?

**DOI:** 10.1007/s00467-019-04271-1

**Published:** 2019-06-25

**Authors:** A.D. Lalayiannis, N.J. Crabtree, M. Fewtrell, L. Biassoni, D.V. Milford, C.J. Ferro, R. Shroff

**Affiliations:** 1 Nephrology Department Great Ormond St. Hospital for Children NHS Foundation Trust and University College London Institute of Child Health, London, UK; 2 Birmingham Women’s and Children’s NHS Foundation Trust, Birmingham, UK; 3 University Hospitals Birmingham NHS Foundation Trust, Birmingham, UK

**Keywords:** Chronic kidney disease (CKD), Dual-energy X-ray absorptiometry (DXA), Peripheral quantitative CT (pQCT), Bone biopsy, Bone mineralisation

## Abstract

Mineral and bone disorder in chronic kidney disease (CKD-MBD) is a triad of biochemical imbalances of calcium, phosphate, parathyroid hormone and vitamin D, bone abnormalities and soft tissue calcification. Maintaining optimal bone health in children with CKD is important to prevent long-term complications, such as fractures, to optimise growth and possibly also to prevent extra-osseous calcification, especially vascular calcification. In this review, we discuss normal bone mineralisation, the pathophysiology of dysregulated homeostasis leading to mineralisation defects in CKD and its clinical consequences. Bone mineralisation is best assessed on bone histology and histomorphometry, but given the rarity with which this is performed, we present an overview of the tools available to clinicians to assess bone mineral density, including serum biomarkers and imaging such as dual-energy X-ray absorptiometry and peripheral quantitative computed tomography. We discuss key studies that have used these techniques, their advantages and disadvantages in childhood CKD and their relationship to biomarkers and bone histomorphometry. Finally, we present recommendations from relevant guidelines—Kidney Disease Improving Global Outcomes and the International Society of Clinical Densitometry—on the use of imaging, biomarkers and bone biopsy in assessing bone mineral density. Given low-level evidence from most paediatric studies, bone imaging and histology remain largely research tools, and current clinical management is guided by serum calcium, phosphate, PTH, vitamin D and alkaline phosphatase levels only.

## Introduction

There is a growing awareness that mineral dysregulation in chronic kidney disease (CKD) is closely linked to abnormal bone pathology, and that this in turn, may potentially lead to extra-skeletal calcification. The KDIGO (Kidney Disease Improving Global Outcomes) have proposed the broad and encompassing term *chronic kidney disease-mineral and bone disorder* (CKD-MBD) to describe this clinical entity [1]. MBD is the triad of biochemical abnormalities (of calcium, phosphate, parathyroid hormone (PTH) and 1,25-dihydroxyvitamin D), bone abnormalities (short stature, reduced mineralisation and increased risk of fractures) and extra-skeletal calcification [2, 3]. Mineral dysregulation, leading to bone demineralisation, plays a causal role in increased bone pain, deformities and fracture risk in childhood CKD [4, 5].

Assessing bone health should be a key element in the clinical assessment of children with CKD, but the tools available to clinicians are not well researched and have many shortcomings. A detailed assessment of bone health and bone mineralisation in particular requires a combination of biochemical measures, imaging techniques and in some instances, bone biopsy. However, serum biomarkers may not be reflective of the true state of bone turnover or mineralisation and imaging techniques have several limitations and are not widely available. Bone biopsies are considered to be highly invasive and rarely performed in children.

In this review, we focus on the techniques available to the clinician to assess bone mineralisation by imaging methods, histology and biomarker studies. We examine their utility in the clinical setting and discuss relevant recommendations from international guidelines. Although bone mineralisation is best assessed by bone histomorphometry [6, 7], radiological techniques are also used to assess bone mineral density (BMD) as a proxy in clinical practice and some studies. Given the low level of evidence from most clinical studies, these techniques are largely reserved for research, and current clinical management is guided by serum calcium, phosphate, PTH, vitamin D and alkaline phosphatase levels only.

## Normal bone mineralisation in the growing skeleton

**Fig. 1:**
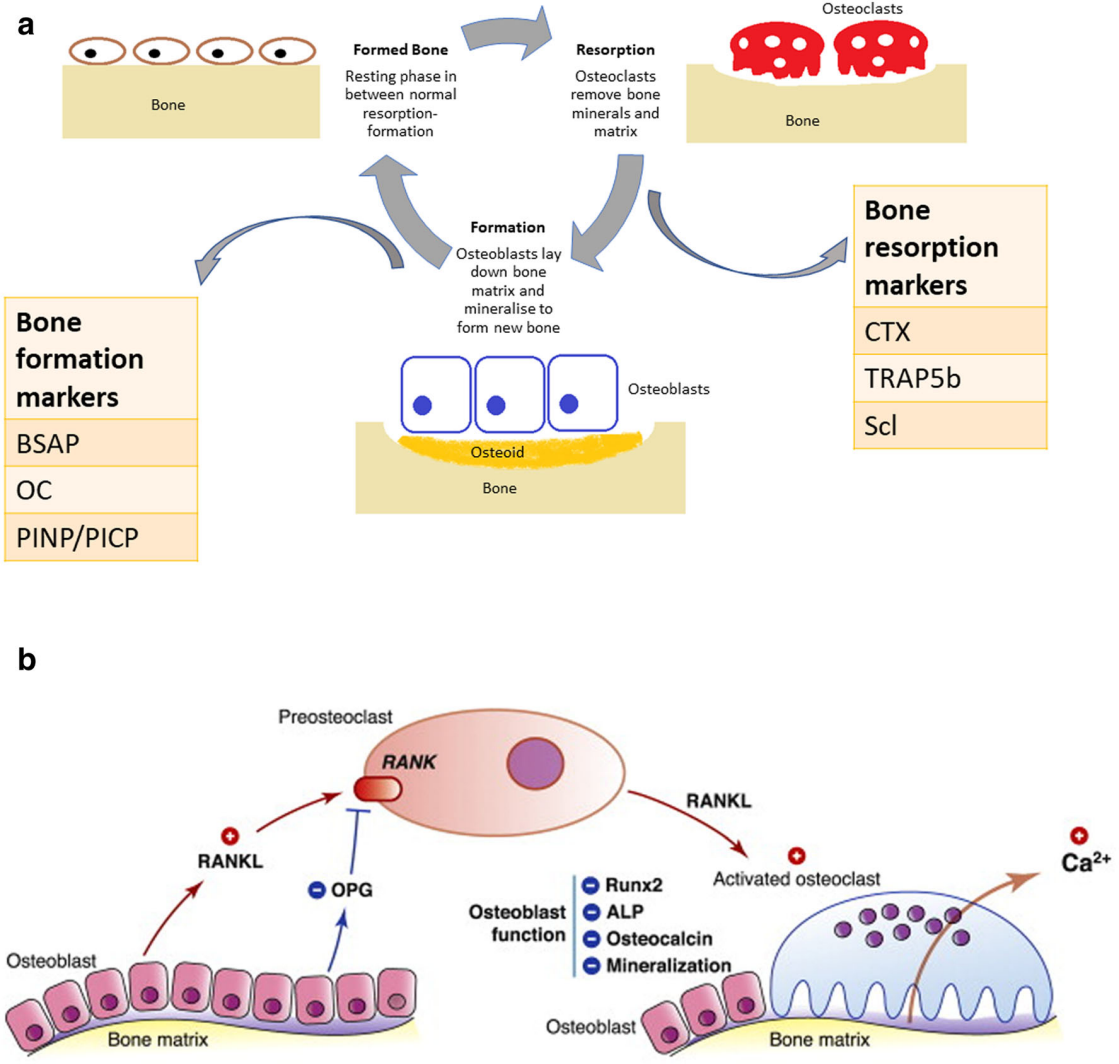
**a** Remodelling of bone is controlled by osteoblasts and osteoclasts. Bone formation happens through organic matrix formation (osteoid), that gets mineralised to form bone, and finally undergoes remodelling by resorption and reformation. Calcium and phosphate form hydroxyapatite that deposits in the extracellular compartment, between collagen fibres. Osteoclasts are responsible for bone resorption, removing bone minerals and matrix. Certain biochemical markers reflect bone turnover and bone cell activity. Bone regulators can be grouped broadly into bone turnover factors (e.g. PTH, sclerostin) and bone cell activity indicators (bone formation, e.g. bone-specific alkaline phosphatase (BSAP), osteocalcin (OC), procollagen type I N propeptide (PINP), procollagen type I C propeptide (PICP); bone resorption, e.g. carboxyterminal cross-linking telopeptide of bone collagen (CTX), tartrate-resistant acid phosphatase (TRAP5b)). **b** Bone resorption is activated by the RANK-RANKL-OPG pathway, which regulates osteoclast differentiation and activation. Osteoclast precursors express RANK, which is activated by its ligand, RANKL, produced by osteoblasts and osteocytes. Osteoprotegerin (OPG), also a product of osteoblasts and osteocytes, is a decoy receptor for RANKL, neutralising the osteoclastic function activated by the RANKL-RANK complex. Thus, the RANKL/OPG ratio is an important determinant of bone mass as it affects mineralisation, alkaline phosphatase, Runx2 and osteocalcin which reflect osteoblast differentiation and bone formation rate. Figure adapted from Charoenphandhu et al. [117]

Bone development involves a process of organic matrix formation (osteoid), that is mineralised to form bone, and finally undergoes remodelling by resorption and reformation, old bone being replaced continuously by new bone (Figs. 1a and b) [8, 9]. Remodelling of bone is controlled by osteoblasts and osteoclasts. Osteoblasts secrete procollagen during bone formation, and the end peptides are cleaved off during matrix formation to produce type 1 collagen. This structure serves as a scaffold around which mineralisation occurs [10]. Phosphates are released locally from the matrix vesicles in the osteoid. Calcium from the extracellular compartment then forms hydroxyapatite with the phosphates [11]. These crystals are also comprised of small amounts of sodium, magnesium and carbonate [12]. When the newly formed crystals are no longer soluble, they deposit in the form of hydroxyapatite in the vesicles. If there is sufficient phosphate and calcium available, hydroxyapatite formation continues to fill the spaces between the collagen fibres [13]. New bone formation happens by modelling and longitudinal growth at the growth plates by chondrocytes which are located between the epiphyses and metaphyses of long bones. The growth plates gradually move further away from the bone centre, producing elongation of the bone [14, 15]. Growth hormone is key in this process by driving the differentiation of chondrocytes to osteogenic cells, increasing cellular proliferation, increasing insulin-like growth factor 1 (IGF1) that stimulates the expansion of the chondrocytes and increasing deposition of type 1 collagen leading to further bone growth [14].

Bone mass, on the other hand, depends on the balance between bone resorption and formation. Bone modelling produces growth of the skeleton and changes in bone shape in response to mechanical forces [16]. This is the predominant driving force in early life. Remodelling renews bone and maintains mineral homeostasis. It repairs bone damage by resorbing old bone and forming new bone. In the growing skeleton, remodelling occurs very rapidly with a positive balance to account for mineral deposition and skeletal growth [17, 18]. Both phases of bone metabolism—modelling and remodelling—are crucial in order to maintain good bone strength and prevent fractures.

## Calcium requirements for normal bone mineralisation

Peak bone mass (PBM) is defined as ‘the amount of bone gained by the time a stable skeletal steady state has been attained during adulthood’ [19]. This incorporates and influences bone strength, which is derived from the structural properties provided by mass, density, microarchitecture and geometry of the bone. In a prospective study of 125 healthy girls over 8 years, girls who sustained at least one fracture (*n* = 42, cumulative incidence 46.6%) were more likely to have lower bone mass by DXA [20]. The US National Osteoporosis Foundation position statement on peak bone mass development gives high-level evidence for physical activity and calcium intake on bone accretion, especially during late childhood and peripubertal years [19]. Normal bone mass accrual during growth is extremely important in preventing fractures in later life [19]. This is not surprising given that the mineral composition of bone accounts for two-thirds of its dry weight [12].

The timing of PBM accrual varies between the sexes and by the skeletal site. The Saskatchewan Paediatric Bone Mineral Accrual Study followed 164 children aged 8–14 years into their early 30s with dietary information and whole body DXA scans [21, 22]. They showed that peak height velocity (PHV) was reached by 11.8 and 13.5 years for females and males, respectively, but PBM (highest BMC by total body DXA) was attained 7 years later. Bone development in terms of growth and length stopped around 5 years after PHV, followed by a plateau of bone mineralisation 2 years later (18.8 and 20.5 years for females and males respectively). However, at the lumbar spine and hips, in particular, PBM was achieved 5 years after PHV [22]. Previous cross-sectional studies had suggested that PBM is achieved even later than this. Recker et al. showed that in 156 healthy adult women, the mineral accrual stopped by 28.3 to 29.5 years of age [23]. Lin et al., studying 300 healthy females aged 6 to 32 years, found that bone mineral content (BMC) was highest in this cohort in the early twenties (23.0 ± 1.4 years), but increases in BMC were seen into the early thirties [24]. In summary, approximately 25% of PBM is formed around the 2-year interval of PHV which happens in adolescence, but PBM is likely achieved by the end of the second or early third decade of life [22].

More than 99% of the calcium in the body is stored in the skeleton. The calcium content of the skeleton increases from 25 g at birth to around 1000 g in adulthood. This is the reason why the growing skeleton of children has a higher demand for calcium compared to adults [25]. Adequate dietary calcium intake during childhood and puberty is necessary for skeletal development and for attaining peak bone mass. Matkovic et al. published an aggregated report of 519 calcium balance studies performed on participants from birth to 30 years old. In every age group, calcium balance positively correlated with calcium intake from diet or supplements. The highest calcium requirement was in the first year of life (503 ± 91 mg/day) and during pubertal growth (396 ± 164 mg/day), dropping thereafter to normal adult requirements (114 ± 133 mg/day) [25]. This implies higher intestinal calcium absorption and uptake by the growing skeleton in childhood and adolescence [25].

Studies have also associated oral dietary calcium intake with bone growth and mineral accrual in childhood [26, 27]. Bonjour et al. showed that daily consumption of a high calcium intake led to a greater increase in radial and femoral BMD in pre-pubescent girls [28]. Similarly, Cadogan et al. demonstrated that an increased intake of calcium (through milk) over 18 months resulted in a significant increase in BMD (9.6% vs 8.5%, *p* = 0.017) and BMC (27.0% vs 24.1%, *p* = 0.009) by DXA in 82 12-year-old girls [29]. A randomised trial of 354 adolescent girls showed that increased calcium intake translated into significantly higher radial and total body BMD on DXA scan over the 7-year follow-up [30]. Abrams et al. using a calcium isotope technique demonstrated that increased dietary calcium absorption was the main driver of net calcium retention in infants and adolescents [31, 32]. The higher calcium requirements in children are also reflected in higher normal values for serum total calcium in children, particularly during periods of active growth in infancy, and reach adult levels by 5 years of age. The (US) National Osteoporosis Foundation’s position statement review found that 90% of randomised control trials using calcium supplement pills showed a statistically significant effect on BMD and/or BMC accrual. This was especially seen in children with a lower calcium intake at baseline [19]. As bone mineral accrual in infancy, childhood and adolescence is closely linked with calcium intake and particularly oral calcium absorption [26, 27], any condition which disrupts this mechanism may lead to poor mineralisation [19].

## Abnormal bone mineralisation in CKD

**Fig. 2:**
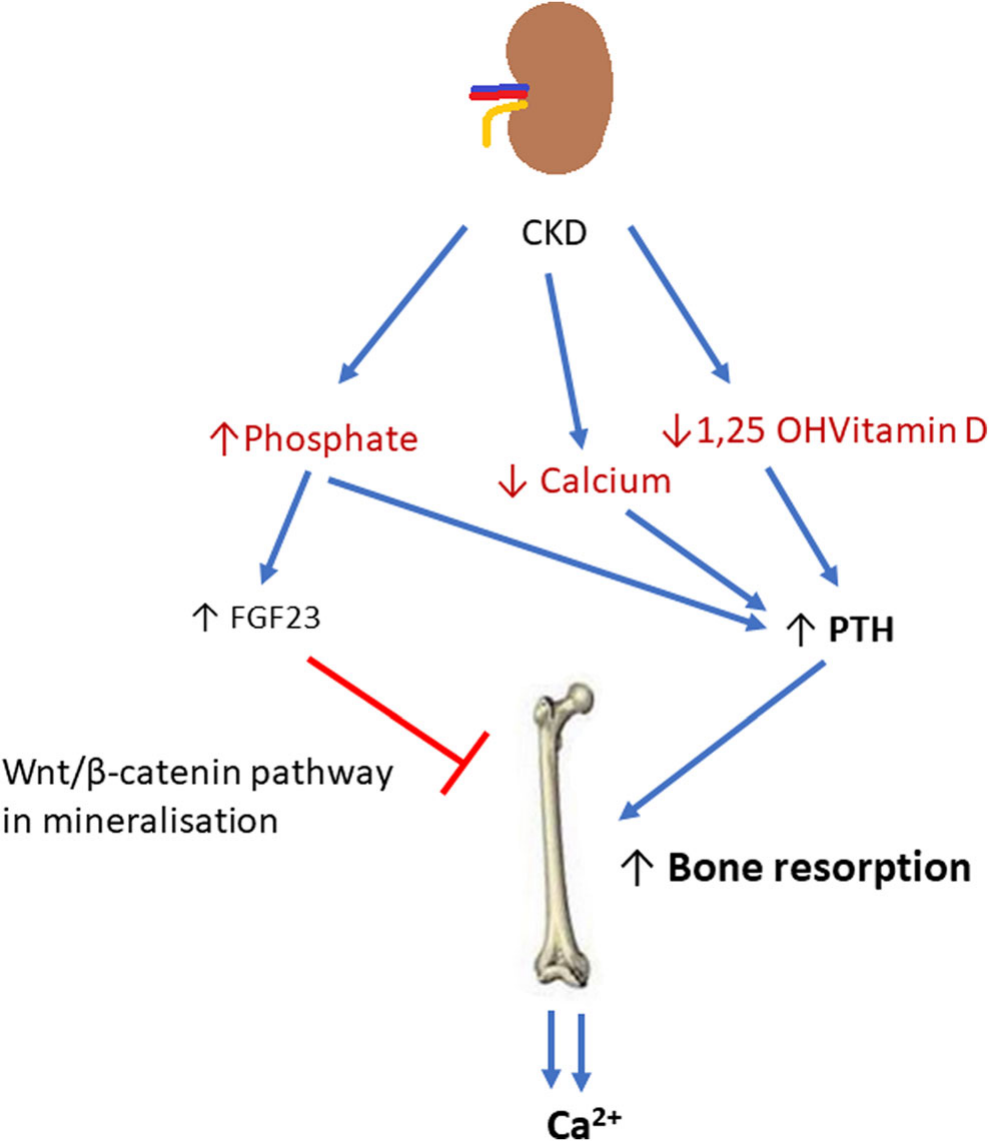
In chronic kidney disease (CKD), hypocalcaemia, low 1,25 OH vitamin D levels and hyperphosphataemia develop. In an attempt to increase phosphaturia, and thus decrease serum phosphate levels, FGF23 production increases. Raised FGF23 may directly inhibit Wnt signalling pathways which are needed in bone mineralisation. Low 1,25OHVitD and low serum calcium lead to increased PTH production. This in turn causes increased bone turnover with the aim of restoring normocalcaemia, by mobilising calcium out of bone. The reduced production of active vitamin D from the kidneys perpetuates hypocalcaemia further fuelling this cycle. This demineralisation affects bone quality as a whole leading to an increased risk of fractures and decreased bone strength

As the glomerular filtration rate reduces, phosphate excretion is impaired [33]. The reduced 1-alpha-hydroxylation of 25-hydroxyvitamin D from the failing kidneys leads to reduced intestinal absorption of calcium and results in hypocalcaemia. Hypocalcaemia stimulates increased PTH production by the parathyroid glands and elevated phosphate levels stimulate fibroblast growth factor 23 (FGF23) secretion by osteocytes (Fig. 2) [34–36]. These compensatory pathways aim to increase phosphaturia and mobilise calcium out of its reservoir in the bone. PTH causes increased phosphaturia by acting on the renal phosphate sodium co-transporter and increases bone resorption [37], releasing calcium, which affects overall bone mineralisation [38–40]. FGF23 may also directly inhibit Wnt signalling pathways which are needed in bone mineralisation [41]. This high resorption state and demineralisation leads to an overall degradation of bone architecture, decreased BMD and increased fracture risk [10, 42].

## The burden of MBD in childhood CKD and the resulting fracture risk

Chronic kidney disease affects bone modelling, remodelling and growth. Poorly controlled CKD leads to reduced bone mass accrual and accelerated bone loss. These alterations occur early in the course of CKD [43] but the entire and complete processes by which these abnormalities of skeletal mineralisation occur are not entirely clear [44]. Bone strength is determined by the bone mass and bone quality. It is affected by many factors including sex hormones, BMD, microarchitectural organisation, geometry and size. Cortical thickness and mineralisation are major contributors to overall bone strength [45, 46]. CKD in childhood affects mineral homeostasis and the normal process of mineral accrual and deposition in the bone, thereby affecting bone mass, architecture and strength [2, 15].

Children with CKD are reported to have bone pain, limb deformities, short stature and a three-fold higher fracture incidence compared to their healthy peers [43, 47]. Borzych et al., using the clinical registry of the International Paediatric Peritoneal Dialysis Network (IPPN), showed that clinical symptoms or radiological signs of bone disease were present in 15% of 890 children and adolescents. These included radiological signs of renal rickets and osteopenia but also limb deformities and pain [33]. Fifty percent of children with CKD will not attain their genetically pre-determined height and remain significantly shorter [48].

The CKiD study (CKD in Children) has recently evaluated the burden of fractures in a large cohort of children. The reported fracture rates of 395/10,000 person-years for males and 323/10,000 person-years for females were 2.4- and 3-fold higher, respectively, than gender-specific rates of 162/10,000 person-years and 103/10,000 person-years reported in a large population-based study of fracture epidemiology in healthy children and adolescents [38], and exceeding those reported in over 12,000 adult haemodialysis patients [38, 49]. The independently linked factors to higher fracture rates were baseline walking difficulty, Tanner stages of pubertal development 4–5, greater height *Z*-score, higher PTH levels and competitive sports participation. The only possible protective factor was phosphate binder use which afforded a 63% lower fracture risk; of note, 82% of patients in this study were on calcium-based phosphate binders, suggesting that improved phosphate control or the calcium absorption from the binder may afford some protective benefit [38, 43].

Lower BMD measured by peripheral quantitative computed tomography (pQCT) was identified as a significant predictor of fracture risk in a study of 170 children and young people up to 21 years old in CKD stages 2–5 and on dialysis. Lower serum calcium levels were independently associated with lower cortical volumetric BMD *Z*-scores. Over a 1-year follow-up in 89 children, a change in the cortical BMD *Z*-score positively correlated with baseline calcium (*p* = 0.008) and increase in calcium (*p* = 0.002) levels, particularly in growing children. Of these participants, 6.5% suffered some form of fracture during the study’s 1-year follow-up (incidence 556/10,000 person-years). Notably, lower cortical BMD *Z*-score predicted future fractures: the hazard ratio for fractures was 1.75 (95% CI 1.15–2.67; *p* = 0.009) per SD decrease in baseline BMD. The fracture sites were the clavicle, tibia, foot, toes and radius. These fractures were sustained in low-impact traumas, such as exercise and falls. Independent risk factors attributed to fracture risk were any period of rapid growth in adolescence, lower calcium and vitamin D (25(OH)D) levels as well as a higher PTH at baseline. All were associated with lower cortical BMD scores [43].

The cumulative burden of MBD that develops during the pre-transplant period may be further exacerbated after transplantation. Children who have received any solid-organ transplant had a 6-fold higher incidence of fractures overall, but particularly vertebral fractures (160-fold) compared to healthy peers in a 5-year follow-up period [50, 51]. Interestingly, a reduced lumbar BMD *Z*-score was observed in 17% of the participants at the time of transplantation [51]. Abnormalities in mineral metabolism also persist after transplantation; in a registry study of 1237 European children, 19% had hypocalcaemia and 40% had a high PTH 3 years after transplantation (interquartile range 1.1–6.2 years) [50].

Mineral and bone disorder in childhood has long-lasting consequences into adulthood. A study of 249 young adults with the onset of end-stage renal failure pre-adolescence and followed into adulthood showed that 37% had symptoms of bone disease (deformities, bone pain, aseptic bone necrosis and atraumatic fractures), 18% were disabled by bone disease and 61% had severe growth restriction [47, 52]. The United States Renal Data System reports that the relative risk of hip fracture was highest in young adults (< 45 years old) on haemodialysis, and hip fractures were associated with a more than 4-fold increase in mortality in dialysis patients compared to healthy age-matched peers [53–55].

## Techniques for assessing bone health

Assessing bone health with a view to predicting and preventing fractures in children with CKD is challenging. Examining bone turnover, density and mineralisation in a non-invasive way that does not involve radiation are not possible. The current tools available for assessing bone turnover and density include bone histomorphometry, blood biomarkers and imaging techniques. We discuss these in turn.

### Bone biopsy

Bone biopsy is considered the gold standard for bone assessment and allows assessment of the dynamic process of bone formation and resorption. The histologic findings are categorised in terms of bone turnover, mineralisation and volume (the ‘TMV’ classification) [56]. Bone biopsy is an invasive procedure and only a handful of adult and paediatric nephrology centres around the world perform this in clinical practice or for research purposes. The procedure requires an anaesthetic and dosing with tetracycline, which has UV fluorescence properties, at two different time intervals to ‘label’ the bone for assessment of the dynamic features of bone formation [57].

A review of five bone biopsy studies, performed in a total of 172 children on HD or PD, showed that 14–37% showed normal bone histology, with 22–43% showing low bone turnover. High PTH levels were able to identify high bone turnover states, but lower PTH levels were not able to distinguish between low and normal bone turnover [58]. A detailed discussion of bone histomorphometry studies and correlations with biomarkers is presented in the section below.

### Serum biomarkers of bone formation and resorption

Regulators of bone mineralisation and turnover can be measured in the blood or urine and reflect the metabolic activity of bone cells (Fig. 1b). They are usually grouped into:

i. Bone formation markers: Bone-specific alkaline phosphatase (BSAP), osteocalcin (OC), procollagen type I N propeptide (PINP), procollagen type I C propeptide (PICP). These markers are products of osteoblast metabolism and activity and reflect the production of type 1 collagen, the structure that serves as a scaffold around which mineralisation occurs [10], and accounts for more than 90% of the organic component of the bone matrix [12]. BSAP reflects bone formation rate [59]. In healthy children, BSAP is associated with age, and gender [60], increasing at the start of puberty, associated with height velocity and higher in boys [60].
ii. Bone resorption markers: Carboxyterminal cross-linking telopeptide of bone collagen (CTX) and tartrate-resistant acid phosphatase (TRAP5b). These markers are mainly by-products of type 1 collagen breakdown [10].
iii. Osteocytic markers: Phosphate-regulating gene with homologies to endopeptidases on the X chromosome (PHEX), dentin matrix protein-1 (DMP1), matrix extracellular phospho-glycoprotein (MEPE), sclerostin and FGF23 that can regulate both osteoblastic and osteoclastic activity [61].

All these biomarkers have been researched, drawing correlations with bone histology, imaging and fracture outcomes, but only PTH and BSAP are considered useful adjuncts to calcium and phosphate measurements in clinical practice.

In bone histomorphometry studies, serum PTH has been associated with both bone mineralisation and turnover (Table 1). However, PTH can remain within normal levels in the early stages of CKD, despite bone biopsy studies showing that almost a third of children in CKD stage 2 have poor bone mineralisation [62, 63]. In 2010, Bakkaloglu et al. reviewed bone biopsies of 161 children on PD and identified mineralisation abnormalities in 48% of all patients. Abnormal mineralisation was found in 58% of participants with high bone turnover, 38% with normal turnover and 29% with low turnover. Serum PTH and alkaline phosphatase correlated with bone turnover (PTH: *r* = 0.61, *p* < 0.01; alkaline phosphatase: *r* = 0.51, *p* < 0.01) and serum calcium was inversely related to mineralisation (*p* < 0.01) but not bone turnover. In any turnover state, higher PTH values and lower calcium values associated with abnormal mineralisation [62]. The authors demonstrated that when both PTH and alkaline phosphatase levels were within 2xULN normal bone turnover and normal mineralisation was seen [62].

**Table 1:** Summary of studies comparing parathyroid hormone levels with bone biopsy data. Some notable studies that have correlated parathyroid hormone (PTH) with bone biopsy findings (Pubmed search strategy: All English language papers). *PD*, peritoneal Dialysis; *HD*, haemodialysis; *Ca*, calcium; *ALP*, Alkaline Phosphatase

Authors, year	Population (*n*)	Age of population (years)	Key findings on bone biopsies	Correlations with PTH	Limitations	Comments
Salusky et al., 1988 [117]	PD (44)	6–18	Normal histology in 16% Osteitis fibrosa in 39% Aplastic lesions in 11% Osteomalacia in 9%	Bone formation rate and larger resorption areas correlated with PTH (*p* < 0.001) PTH values were 2–3× higher in osteitis fibrosa patients	Study prior to TMV criteria Aluminium hydroxide main phosphate binder	Focus of study primarily on aluminium staining—as aluminium hydroxide used as main type of phosphate binder.
Mathias et al., 1993 [118]	HD (21)	16–19	High-turnover disease in 38% Osteitis fibrosa in 23% Adynamic bone in 28%	Bone formation rate correlated with PTH as well as resorption areas (*p* < 0.001).	Study prior to TMV criteria Aluminium hydroxide main phosphate binder	PTH also correlated inversely with serum Ca levels (*p* < 0.001)
Goodman et al., 1994 [119]	PD (14)	13–14	Before calcitriol: osteitis fibrosa in 79% After calcitriol: normal in 43% Adynamic in 43% Osteitis fibrosa in 7% Mixed in 7%	A PTH of below 200 pg/mL was strongly suggestive of adynamic bone disease.	Small number of patients	Aim of study was to look at effect of intermittent calcitriol therapy over 12 months on bone biopsy indices.
Salusky et al., 1994 [65]	PD (55) (68 bone biopsies)	8–19	Osteitis fibrosa in 50% Mild hyperparathyroidism in 9% Adynamic bone lesions in 22% Normal in 19%	High PTH values strongly correlated with osteitis fibrosa lesions vs mild, adynamic or normal histology (*p* < 0.001)		PTH > 200 pg/mL and Ca < 10 mg/dl was 85% sensitive and 100% specific for high-turnover lesions. PTH < 200 pg/mL 100% sensitive, 79% specific for adynamic bone lesions
Yalçinkaya et al., 2000 [120]	PD (17)	7–20	High-turnover disease in 47% Low turnover disease in 29% Mixed in 24%	High PTH values were significantly correlated to high-turnover disease (*p* < 0.01) vs low turnover	Small number of patients	Mean serum Ca levels higher in low-turnover group vs high-turnover group (*p* < 0.001) Serum PTH > 200 pg/mL was 100% sensitive and 66% specific in identifying high turnover.
Ziólkowska et al., 2000 [121]	HD (21), PD (30)	7–15	Adynamic bone disease in 27% Normal bone in 37% Osteomalacia in 2% Hyperparathyroidism in 24% Mixed lesions in 10%	Higher PTH significantly correlated with high-turnover disease vs adynamic or normal bone.		Serum PTH > 200 pg/mL: 75% sensitive and 95% specific for identifying high-turnover disease In patients with normal bone turnover, 69% had PTH level of 50–150 pg/mL
Waller et al., 2008 [66]	Pre-Tx (11)	7–16	Low bone turnover disease in 18% Mixed lesions in 27% Hyperparathyroidism in 36%	PTH > 3× ULN associated with high turnover Normal range PTH associated with low turnover	Small number of patients	
Bakkaloglu et al., 2010 [62]	PD (161)	0–20	Low turnover in 4% Normal turnover in 39% High turnover in 57% Abnormal mineralisation in 48%	Higher PTH significantly correlated with high-turnover disease vs low turnover or normal bone.		For any level of turnover, PTH was higher if mineralisation defects were present (*p* < 0.01). PTH < 400 pg/mL and ALP < 400 IU/L provided the highest prediction of normal bone turnover and mineralisation
Wesseling-Perry et al., 2012 [63]	CKD2-5 (52)	2–21	High bone turnover in: 13% with CKD3 29% with CKD 4/5 Defective mineralisation in: 29% with CKD2 42% with CKD3 79% with CKD4/5	PTH was elevated in 36% of patients with CKD2, 71% with CKD3, and 93% with CKD4/5		PTH was directly linked to poor mineralisation (*p* < 0.05)
Carvalho et al., 2015 [122]	PD (22)	2–16	High bone turnover in 54% Low bone turnover in 23% Normal turnover in 23%	PTH values higher in patients with high bone turnover (*p* < 0.05) and mineralisation (*p* < 0.01)	Small number of patients	Bone turnover correlated with alkaline phosphatase also (*p* < 0.01)

Furthermore, PTH does not discriminate effectively between low- and high-turnover bone disease, in the ranges where most patients’ PTH values are found (100–1000 ng/L) [64]. Salusky et al., looking at biopsies of 55 children, found that serum calcium levels were higher in patients with adynamic bone or normal bone than in those with high PTH values. On the other hand, serum phosphate, alkaline phosphatase and PTH levels were higher in patients with osteitis fibrosa. The combination of a high serum PTH and normal calcium value was 85% sensitive and 100% specific for identifying patients with the high-turnover bone disease [65]. In general, all high-turnover disease was associated with high PTH levels (> 3 ULN upper limit normal) whereas adynamic bone disease and normal bone turnover were associated with lower PTH values, but the ability of PTH to distinguish between the two is not strong.

On clinical correlations, maintaining PTH within normal levels up to 2× ULN was associated with good growth [66]. In a study of 556 children aged 6–18 years with CKD (eGFR 10–60 mL/min/1.73 m^2^), BSAP SDS values decreased with declining eGFR and closely associated with PTH [67]. In patients treated with growth hormone, BSAP showed an increase after treatment. BSAP SDS were predictive of a prospective change in height SDS [67]. The International Paediatric Peritoneal Dialysis Network Registry that followed up nearly 900 children on PD found that clinical and radiological symptoms markedly increased when PTH exceeded 300 pg/mL, the risk of hypercalcemia increased with levels below 100 pg/mL and time-averaged PTH concentrations above 500 pg/mL were associated with impaired longitudinal growth [33]. In a prospective study of 171 children with CKD, high PTH was independently associated with a decline in tibial cortical BMD on annual follow-up. [43]. A further study has also correlated higher BSAP and CTX levels were associated with lower cortical BMD [68].

In summary, no biomarker, individually or in combination, is sufficiently robust to diagnose bone mineralisation or turnover defects [69, 70]. Furthermore, biomarkers vary depending on age, gender, pubertal stage, fasting status and circadian rhythms and assays are not always standardised [60, 71]. Larger paediatric studies that correlate bone biomarkers with the gold standard of bone histomorphometry as well as patient-level outcomes such as fractures are required.

### 3. Imaging techniques

#### Dual-energy X-ray absorptiometry

DXA is a tool that is used for evaluating bone density and assessing fracture risk in children and in adults. There is a growing body of literature on the use of DXA in different paediatric diseases [72].

##### Principle

A DXA scanner produces X-rays at 2 different energies, enabling the differentiation of soft tissue and bone [73]. Even at low levels of radiation (4–27 μSv) [74], DXA allows for measurement of bone mineral content (BMC) and projected bone area (BA), from which the areal BMD is calculated as BMC/BA.

##### Technique

Measurements can be made at the spine, hip, forearm or whole body (WB, also described as the total body (TB)) [73]. Common sites in paediatric practice include lumbar spine (LS) and total body less head (TBLH) [75]. In children, LS DXA is particularly useful because vertebrae are mainly trabecular bone, and this site is readily influenced by pathologic processes, due to the rapid bone turnover [72]. The L1 to L4 region is recommended in the posterior-anterior direction. In adults, the recommended site is the femoral neck, as any possible aortic calcification can hinder interpretation of the lumbar region. Changes with growth and variations in development limit the use of the femoral site in pre-pubertal children. Good positioning of the patient by the operator is vital, but subsequently scanning lasts only a few seconds. Imaging can be blurred by motion artefact in very mobile children [76].

##### Information obtained

**Fig. 3:**
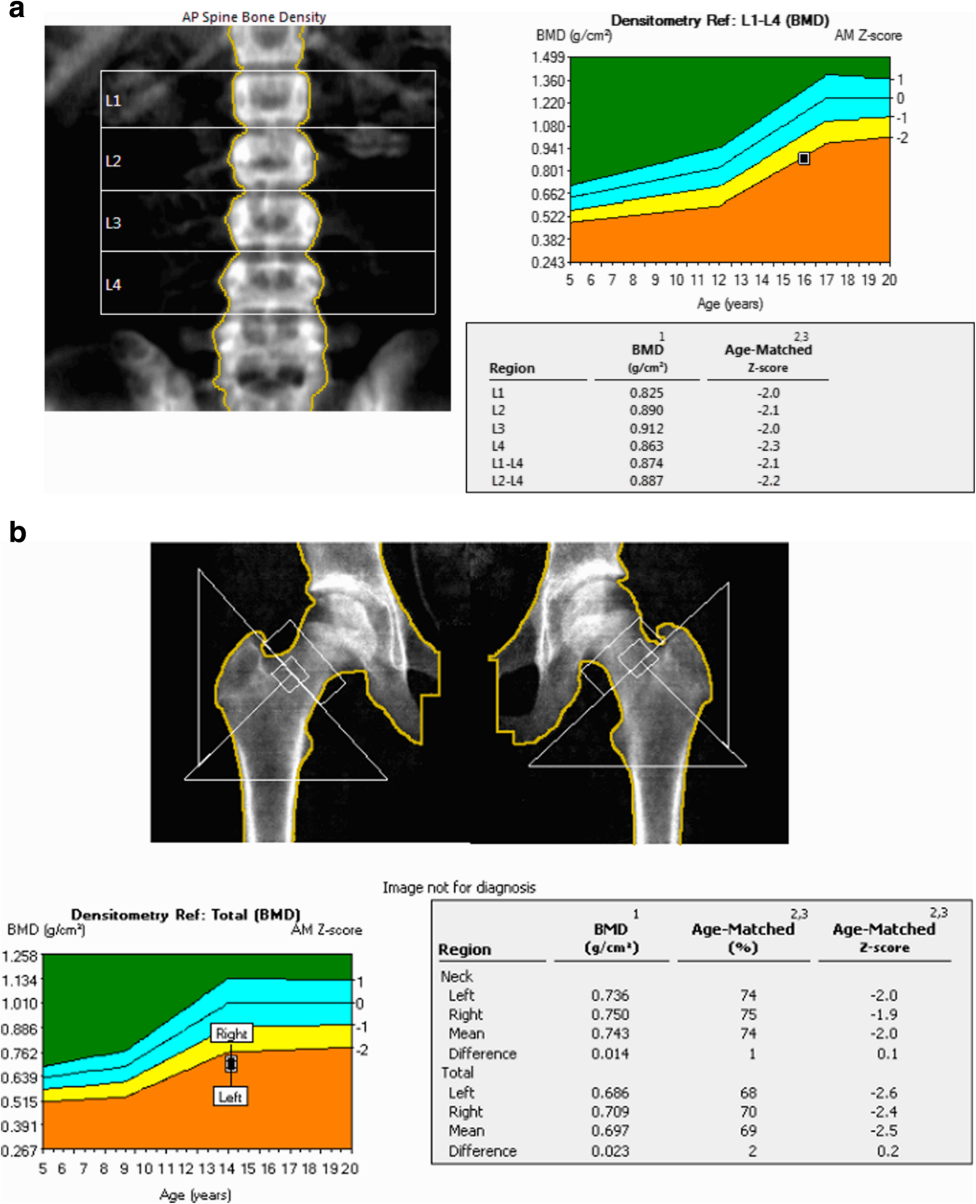
**a** DXA images. This is an example of DXA imaging of the L1-4 spine of a 16-year-old male with chronic kidney disease. His mean L1-4 age-matched *Z*-score is − 2.2. However, when adjusted for his shorter height and poor growth, his BMAD *Z*-score is − 0.8 (the BMAD value is obtained by adding the bone mineral content of the L1-L4 vertebrae and dividing by the total volume of the 4 vertebrae). **b** This is an example of a DXA image of both hips of a 14-year-old girl with chronic kidney disease on home nocturnal haemodialysis. Her mean age-matched *Z*-score for both hips is − 2.5

DXA gives information on BMC (Table 2), measured in grams (g). This is produced automatically by the software and can be normalised for the child’s height or bone area. BMD and content of mineral in a projected area of bone (g/cm^2^) are automatically derived and expressed in age, sex and ethnicity matched *Z*-scores [77]. If the score is below 2 standard deviation points (− 2 SD), then it indicates an abnormally low BMD for age [75]. Importantly, BMD reflects density in a two-dimensional projection only. Therefore, it may be misleading in shorter children or those with delayed growth, as the projected bone area will be smaller. To overcome this problem, bone mineral apparent density (BMAD) is used to try and estimate the whole bone density in gram per cubic centimetre. A *Z*-score is also the preferred comparator for this measure (Fig. 3a and b).

**Table 2:** Important terminology used when assessing bone. The terminology used when assessing bone. Important distinctions are made between BMC and BMD. The definitions of areal BMD and BMAD ensure that the limitations of DXA scanning in growth-stunted children with CKD are accounted for

Terminology	Definition, units and description
Bone mineral content (BMC)	The amount of mineral found in an area of bone. Calcium is the predominant mineral found in bone. Measured in grams (g)
Bone mineral density (BMD)	Mass of mineral per unit volume of bone This reflects the ratio of bone mass to bone volume. Referred to as cortBMD for the bone cortex and trabBMD for the trabecular bone The ratio of BMC over bone size and thus expressed in g/cm^3^. BMD has been used interchangeably with areal BMD in the literature.
Areal bone mineral density (areal BMD)	A term used within DXA reporting. Mineral mass of the bone, divided by projection area of the X-rays (BMC/BA). The commonest parameter used in assessing bone. Represents a composite of bone size and mass Expressed in g/cm^2^
Bone mineral apparent density (BMAD)	BMAD is obtained by dividing the BMC by the estimated three-dimensional bone volume derived from its two dimensional projected bone area. This is done in children to account for growth and size in assessing bone density. For example, the lumbar spine is assumed to be of cylindrical or cuboidal shape in children when calculating BMAD.

##### Reference data in healthy children

Reference data for lumbar spine (L1-L4) BMAD and areal bone mineral density (areal BMD) in healthy children are available from 3598 healthy 4 to 20 year olds from 7 UK centres [78]. Studies providing reference curves from other datasets, mainly from the USA also exist [79–82]. DXA results should be adjusted for bone size (i.e. growth of the child), consistent with the 2013 International Society of Clinical Densitometry (ISCD) Paediatric official positions [76]. Equations to adjust DXA results for height *Z*-score are available [83]

##### Relative advantages and disadvantages

Table 3 lists the main advantages and shortcomings of DXA in children. The greatest drawback of the DXA technique is that it assesses a three-dimensional structure (e.g. spinal vertebral body) as a two-dimensional image. Trabecular and cortical bone are superimposed, so studying the trabecular and cortical BMD separately cannot be done. Given that DXA measures areal BMD (g/cm^2^), it can underestimate volumetric BMD (g/cm^3^) in children with short stature [83] and overestimate BMD in a tall child [73]. Therefore, it is vital *Z*-scores are adjusted for poor growth especially in CKD [84]. Serial DXA scanning in growing children can be particularly challenging.

**Table 3:** Advantages and disadvantages of DXA imaging in children. The main advantages and disadvantages of DXA use in assessing bone in childhood CKD

Advantages	Disadvantages
Low radiation dose (4–27 μSv)	Two-dimensional image—cannot distinguish between cortical and trabecular bone
Evaluation of body composition is possible	Assesses areal BMD in g/cm^2^, not density in g/cm^3^
Operator independent—serial follow-up and standardisation across sites possible	Underestimates BMD in children with poor growth
Widely available	Does not evaluate the microarchitecture of bone
Reference data standardised for age, sex, race and height adjusted standard deviation scores (SDS) available	

##### Studies in CKD

In adults with CKD, there is convincing evidence that lower BMD by DXA indicates an increased fracture risk. Yenchek et al. studied BMD by the femoral neck and total hip DXA in 2754 older adult individuals and showed that lower femoral neck BMD was associated with a higher fracture risk regardless of CKD status (hazard ratio 2.69, 95% confidence intervals 1.96–3.69) [85]. In a longitudinal study by Iimori et al., adult participants with the lower total hip (*p* = 0.0006) or whole body (*p* = 0.006), BMD scores were more likely to have new fractures [86]. Two further cross-sectional studies by Nickolas et al. have shown associations between hip BMD and fracture history in adults with CKD [87, 88].

There are a few notable studies that have used DXA to determine BMD in the context of childhood CKD. Few explore associations with mineral dysregulation and fracture risk in children (Table 4). A study of 40 children both with pre-dialysis CKD and on dialysis showed that all had decreased BMD [89]. Waller et al. used lumbar DXA to assess BMD in a paediatric cohort of 64 patients with CKD, whilst studying biochemical markers and found that maintaining normal calcium, phosphate and PTH concentrations was associated with normal lumbar BMD and growth [90]. Griffin et al. used DXA and pQCT to assess BMD in 88 children with CKD stages 4–5 and compared the scores to 650 healthy participants aged 5–21 years old. They demonstrated that adjusting for lower height Z-scores in the CKD population results in increased BMD *Z*-scores in the lumbar spine and whole body DXA scans. This accounts for poor growth in this population, thereby avoiding overestimation of bone deficits [84].

**Table 4:** Summary of studies using DXA scanning in children with CKD. Some notable studies that have used DXA to study BMD in children with CKD (Pubmed search strategy: All English language papers from 2000 to 2018).*LS*, lumbar spine; *TH*, total hi; *WB/TB*, whole body/total body; *WBLH/TBLH*, whole body less head/total body less head; *Tx*, transplantation; *BMD*, bone mineral density; *iPTH*, intact parathyroid hormone; *P*, phosphate; *HD*, haemodialysis; *PD*, peritoneal dialysis; *Ca*, ,calcium; *iCa*, ionised calcium; *ALP*, alkaline phosphatase; *GC*, glucocorticoid; *25OHD*, 25-hydroxy vitamin D; *rhGH*, recombinant human growth hormone; *PINP*, serum type I procollagen intact amino-terminal propeptide

Authors, year	Population (*n*)	Age of population (years)	DXA	Key findings	Biochemical correlations	Limitations	Comments
Dialysis (haemodialysis and peritoneal dialysis) and chronic kidney disease							
Pluskiewicz et al., 2002 [123]	HD and PD (30: 11 HD, 19 PD)	9–23	LS, TB	Low spine and TB BMD *Z*-scores (− 1.47 and − 1.53). These also correlated with each other (*p* < 0.0001) and with dialysis vintage (*p* < 0.05).	No correlation found between BMD and Ca, iPTH and P. iCa correlated with low spine BMD.	No longitudinal data Small number of participants	
Pluskiewicz et al., 2003 [89]	CKD5, HD and PD (40: 15 CKD5, 9 HD, 16 PD)	7–19	LS, TB	Low spine and TB BMD *Z*-scores in all CKD Population Dialysis vintage correlates with low TB-BMD in dialysis population (*p* < 0.05)	High iPTH correlated with low TB-BMD *Z*-scores in pre-dialysis patients (*p* < 0.05).	A small number of participants	The study compared DXA with QUS also, with QUS parameters lower in CKD population
Bakr, 2004 [124]	CKD5 and HD (65: 21 CKD5, 44 HD)	3–16	LS	61.9% of pre-dialysis children had low LS BMD and 59.1% of HD patients.	LS *Z*-scores of the osteopenic children negatively correlated with P (*p* = 0.004), iPTH (*p* = 0.03), and ALP (*p* = 0.02). There was a positive correlation between LS *Z*-scores and 25OHD	The biochemical analysis only is done in children with low *Z*-scores	
Pluskiewicz et al., 2005 [125]	HD and PD (18: 9 HD, 9 PD)	8–21	LS, TB	Longitudinal data over 2 years showed TB *Z*-score was lower at the end of the study compared to baseline (*p* < 0.05). Spine BMD was lower at the end of the study compared to baseline (*p* < 0.01) in participants without GC use, and those with (*p* < 0.05).	iPTH, Ca, iCa and P did not correlate with skeletal measures.	A small number of participants Comparisons are done in 2 groups; GC use and no GC use	The study compared DXA with QUS. Significant population overlaps with the 2 aforementioned studies, as this study provides the longitudinal follow-up.
Andrade et al., 2007 [126]	HD and PD (20: 6 HD, 14 PD)	4–17	LS	25% had LS *Z*-scores < − 2 SD, but these improved when adjusted for height. 60% of children has the low-bone turnover disease.	No correlations found between bone turnover and Ca, P, PTH or ALP	A small number of participants No comparison of DXA BMD with biochemical findings Limited mineralisation reporting on bone biopsies	LS BMD *Z*-scores improved when adjusted for height. BMD did not correlate with high or low bone turnover
Chronic kidney disease							
van der Sluis et al., 2000 [127]	CKD3-5 (33)	3–12	LS, TB	LS BMD increased with rhGH; ∆SDS 0.72/year (*p* < 0.01) No change was seen with TB BMD	ALP increased in the growth hormone group significantly (*p* < 0.05)	The study aimed at comparing GH use vs no GH use over 2 years	The study compared 18 children with CKD receiving rhGH vs 15 who did not over 2 years.
van Dyck et al., 2001 [128]	CKD 4-5 (10)	2–8	LS, TB	LS and TB BMD *Z*-scores increased after 1 year of rhGH treatment (*p* < 0.01 and *p* < 0.05)	After 1 year of rhGH, there was a significant rise in ALP from 308 μ/L (124 ± 621) to 720 μ/L (226 ± 1067)	The study aimed at comparing BMD before and after 1 year of rhGH treatment. Small cohort	
Waller et al., 2007 [90]	CKD3-5 (64)	4–16	LS	The mean *Z-*score for BMD was normal (*Z*-score = 0.0 (95% CI − 0.29 to 0.28)). Only 8% of the patients had a BMD *Z-*score of less than − 2.0.	BMD *Z-*score did not correlate with any biochemical markers.	Only 2 participants had significantly raised PTH (> 200 pg/mL).	Strict PTH and MBD control in this population shows that maintenance of normal calcium, phosphate and PTH concentrations allows for normal LS BMD and good growth.
Swolin-Eide et al., 2007 [129]	CKD2-5 (16)	4–18	TB, TH, LS	TB and TH BMD increased on average after 1 year (*p* < 0.01). LS Z-scores did not change significantly.	There was a correlation between iPTH and LS BMD. PINP correlated with TB (*p* < 0.05), LS (*p* < 0.01) and TH BMD (*p* = 0.05).	A small number of participants No healthy controls	All biochemical markers were within the normal range. Strict MBD control in this cohort may be the reason that only 44% had BMD *Z*-scores below zero and 38% for LS BMD. Also, the severity of CKD must be factored in; median GFR was 46 (12–74) mL/min/1.73 m^2^.
Swolin-Eide et al., 2009 [130]	CKD1-5 (15)	4–15	TB, TH, LS	Only 5 patients had TB *Z*-scores below 0 at start of the study. On average, LS, TB and TH BMD increased over the study period of 3 years.	Most patients had raised PTH levels (median 95, 23–407 ng/L).	A small number of participants Wide range of GFR, with earlier stages of CKD, included.	Median glomerular filtration rate of 48(8–94 mL/min/1.73 m^2^) may explain why the BMD *Z*-scores were good at baseline.
Griffin et al., 2012* [84]	CKD 4-5 (88)	5–21	LS, TBLH	Adjusting for lower height *Z*-scores in CKD population results in increased BMD Z-scores in LS and TB DXA scans.	LS BMD & TB BMC Z-scores not associated with iPTH or P levels.	No comparison to fracture events Biochemical comparison did not include calcium. Cross-sectional data only No comparison to bone histomorphometry	pQCT showed lower tibial cortical density in CKD, but higher trabecular *Z*-scores.
Post-renal transplant							
Tsampalieros et al., 2014* [91]	Post-renal Tx (56)	5–21	LS, TBLH	Children under 13 years had a significant reduction in LS BMD over 12 months (− 0.65, − 1.16 to − 0.09), *p* = 0.006). Greater GC exposure correlated with greater LS and TBLH *Z*-score reduction. TBLH *Z*-scores were significantly lower in Tx recipients than controls (*p* = 0.02).	iPTH reduction correlated with greater LS *Z*-score BMD reduction.	No comparison to bone histomorphometry No comparison to fracture events	The Pearson correlations between tibia pQCT trabecular volumetric BMD and DXA LS BMD *Z*-scores were 0.45 (*p* < 0.01) and 0.36 (*p* = 0.02) at baseline and 12 months.

*Authors also used peripheral quantitative CT (pQCT) as a comparator

Importantly, a study in 56 children after renal transplantation used both DXA and tibial pQCT to determine fracture risk [91] found that changes in DXA spine-BMD and tibial pQCT trabecular BMD correlated (*r* = 0.47, *p* < 0.01) and changes in whole-body BMC *Z*-scores were associated with changes in tibia cortical area *Z*-scores (*r* = 0.52, *p* < 0.001), but not changes in cortical BMD *Z*-scores. This suggests that the theoretical limitations of DXA in determining cortical and trabecular BMD may not be a major limitation in CKD patients after all.

#### Quantitative computed tomography

##### Principle

QCT is a technique whereby CT images acquired are analysed by specific software to obtain quantitative measures such as volumetric bone mineral density (volumetric BMD) and BMC in any bone compartment [92]. Other measures acquired can be bone cross-sectional area or cortical thickness. Whole body CT scanners can be used, as well as specifically peripheral QCT scanners. The scanners used most commonly are the XCT 2000 and 3000 scanners (Stratec Medizintechnik, Pforzheim, Germany). They use a rotate/translate technology and can produce a 2D slice in around 1 min [74].

##### Technique

pQCT has evolved from the quantitative CT techniques in the 1970s and can now be done rapidly, accurately and peripherally. pQCT provides volumetric and density data in gram per cubic centimetre. It can distinguish between trabecular and cortical bone compartments independent of the size of the subject [73]. Due to this differentiation and because the trabecular bone is more metabolically active, pQCT offers a unique perspective when studying and assessing bone.

**Fig. 4:**
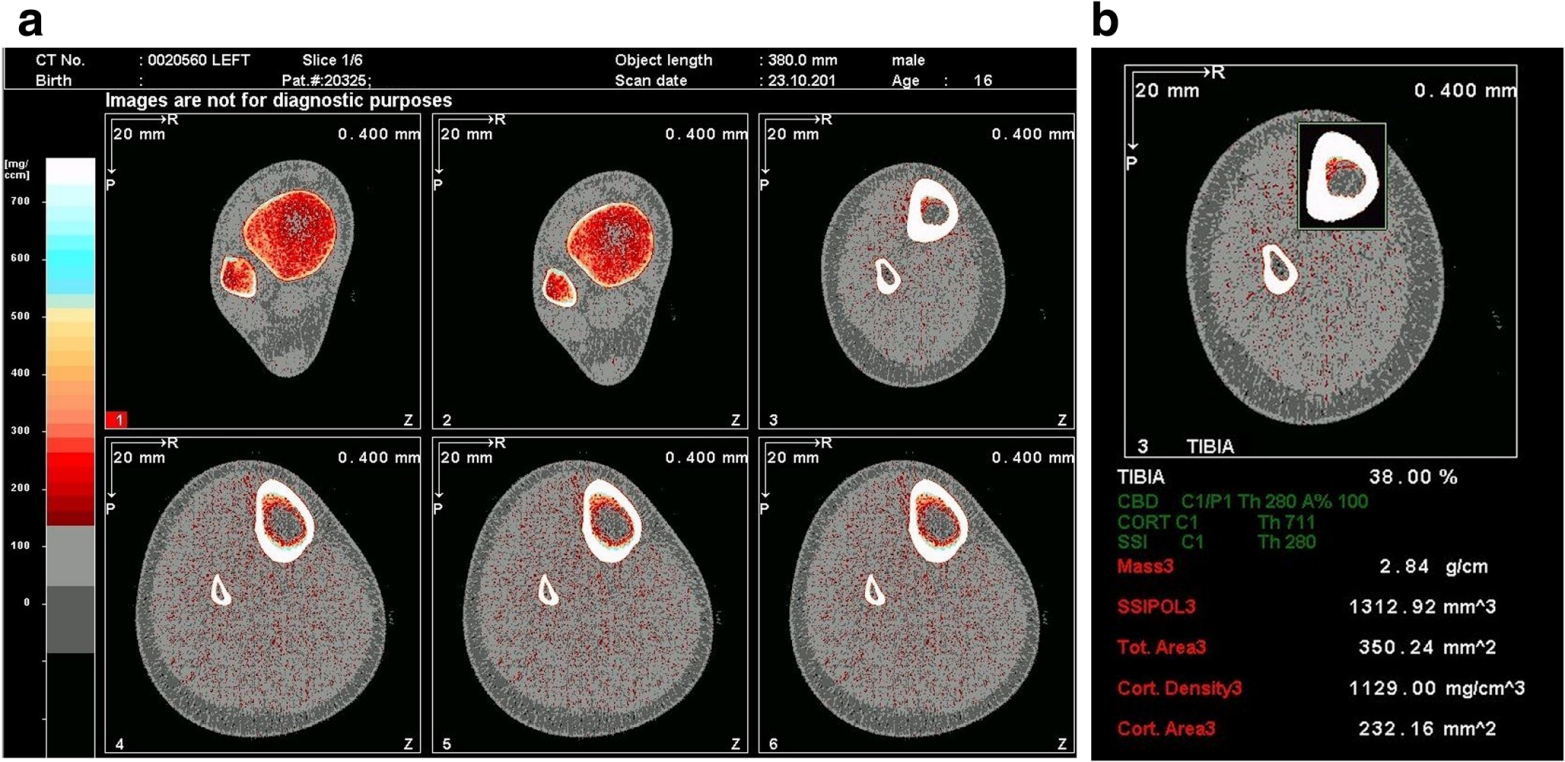
**a** pQCT images. This is an example of pQCT imaging of the left tibia of a 16-year-old male with chronic kidney disease. The images have been obtained at 4 different sites along the tibia. The software then proceeds to automatic analysis of the bone parameters. In this example, the images are from the 3%, 4%, 38% and 66% sites. **b** This is an example of the analysis of the 38% site of the left tibia of a 16-year-old male with chronic kidney disease. In this particular analysis, the total mass, total area, cortical area and cortical density have been given

Performing the scan requires the placement of a reference line on an initial ‘scout view’ image of the distal portion of the limb to be scanned; the software then selects the appropriate slices longitudinally. The percentage distance from the reference line for the images is marked by the operator in a pre-set program. This also relies heavily on the operator’s manual measurement of length from the tibial plateau to the medial malleolus in the lower limb, or from the head of the radius to the styloid process in the upper limb. The reference line must be placed correctly to avoid acquiring images at the growth plates, growth arrest lines or bisphosphonate treatment lines, as these can produce erroneous results (see Fig. 4a and b).

##### Reference data in healthy children

Reference data for children is rather limited but published studies can be used to calculate age-, height- and gender-matched *Z*-scores of the radius or tibia [74]. There is, however, considerable heterogeneity in the literature with regard to the location on a measured limb from which the images are obtained [93–98]. This must be taken into account when choosing a reference database

##### Relative advantages and disadvantages

Table 5 lists the main advantages and shortcomings of pQCT in children. pQCT is largely used in research practice only. The main drawback is the marked heterogeneity in the literature in terms of reference line placement, and where the most appropriate slice location is on the tibia or radius for accurate and reproducible assessments. (Anatomical measurement sites reported include 4%, 20%, 30% and 66% in the radius and 3%, 4%, 14%, 20%, 38% and 66% in the tibia from the distal end of the bone [74, 95]. This heterogeneity means there is a paucity of reference data for age, height and puberty staging for comparison [93–98].

**Table 5:** Advantages and disadvantages of pQCT imaging in children. The main advantages and disadvantages of pQCT use in assessing bone in childhood CKD

Advantages	Disadvantages
Low radiation dose (< 1 μSV)	Operator dependent on placing reference line during scanning
Can distinguish between cortical and trabecular bone	Not widely available, as mainly used in research
Other measures acquired: cross-sectional bone area, cortical thickness	Reference data heterogeneous and not standardised
Volumetric density measured in g/cm^3^	
Independent of patient size, so the height and weight of the patient do not skew results	

##### Studies in children with CKD

In childhood CKD, pQCT has been used successfully to demonstrate the changes in bone demineralisation seen as the disease progresses (Table 6).

**Table 6:** Summary of studies using pQCT scanning in children with CKD. Some notable studies that have used peripheral quantitative CT imaging to study BMD in children with CKD (Pubmed search strategy: All English language papers from 2000 to 2018). *pQCT*, peripheral quantitative CT; *BMC*, bone mineral content; *LS*, lumbar spine; *Tx*, transplantation; *ALP*, alkaline phosphatase; *BSAP*, bone-specific alkaline phosphatase; *β-CTX*, C-terminal telopeptide of type I collagen; *25OHD*, 25-hydroxy vitamin D; *1,25OH*_*2*_*D*, 1,25-hydroxy vitamin D; *Ca*, calcium; *P*, Phosphorus

Authors, year	Population (*n*)	Age of population (years)	pQCT site	Key findings	Biochemical correlations	Limitations	Comments
Dialysis (haemodialysis and peritoneal dialysis) and chronic kidney disease							
Wetzsteon et al., 2011 [68]	CKD 2-5, HD, PD (156: 120 CKD2-5, 36 24 HD, 12 PD)	5–20	Tibia	Trabecular BMD *Z*-scores were inversely associated with age and CKD severity (*p* < 0.001). CKD 4-5 had the lowest cortical BMD (*p* = 0.006).	Greater iPTH (*p* < 0.01), BSAP (*p* < 0.05) and β-CTX (*p* < 0.02), were associated with lower cortical BMD *Z*-scores.	No bone biopsy data No longitudinal follow-up	
Griffin et al., 2012* [84]	CKD 4-5 (88)	5–21	Left tibia	pQCT showed lower tibial cortical density in CKD patients, but higher trabecular *Z*-scores.	No analysis of pQCT vs biochemical markers was made.	No comparison to fracture events. No comparison to bone histomorphometry	Tibial cortical BMC was significantly correlated with TB BMC, and tibia trabecular BMD with LS BMC (*p* < 0.0001).
Denburg et al., 2013 [43]	CKD2-5, HD, PD (171: 109 CKD, 34 HD, 18 PD)	5–21	Tibia	Cortical BMD *Z*-scores were lower in CKD4-5 patients (*p* = 0.002). A greater calcium increase over a year was associated with cortical BMD increases (*p* = 0.002). Increases over a year of iPTH and 1,25OH_2_D were associated with decreases in cortical BMD.	Higher Ca and 25OHD were associated with greater cortical BMD *Z*-scores. The opposite was true for P and iPTH.	No bone biopsy data No ionised calcium measurement	Important to note that 6.5% of participants suffered a fracture over 1 year, and this was associated with lower cortical BMD (HR 1.75, 95% CI 1.15–2.67)
Tsampalieros et al., 2013 [132]	CKD3-5, HD, PD (103: 77 CKD, 16 HD, 10 PD)	5–21	Left tibia	Trabecular BMD did not change significantly over 1 year. Cortical BMD *Z*-scores decreased significantly over 1 year (*p* = 0.02), although baseline *Z*-scores were no different to reference participants (*p* = 0.06).	Higher iPTH values correlated with greater trabecular BMD changes (*p* < 0.001). Baseline cortical *Z*-scores were inversely associated with iPTH levels.	No bone histomorphometry available	
Post-renal transplant							
Tsampalieros et al., 2014* [91]	Post-renal Tx (56)	5–21	Tibia	The Pearson correlations between tibia pQCT trabecular volumetric BMD and DXA LS BMD *Z*-scores were 0.45 (*p* < 0.01) and 0.36 (*p* = 0.02) at baseline and 12 months. The decrease in pQCT trabecular volumetric BMD *Z*-score was significantly greater than the decrease in spine-BMD *Z*-score (− 1.06 ± 1.29 vs. − 0.43 ± 0.77, *p* < 0.01)	pQCT not compared to biochemical markers.	pQCT used mainly as a comparator to DXA imaging.	Important to note that bone density values differed considerably between patients with high- and low-turnover lesions on bone biopsy.

*Authors also used DXA to assess the bone mineral density

A study comparing 156 children with CKD (stages 2 to 5 including 36 on dialysis) to 831 healthy participants (ages 5–21 years) using tibial pQCT showed that iPTH levels above the Kidney Disease Outcomes Quality Initiative (KDOQI) recommended target was associated with increased trabecular BMD *Z*-scores (*p* < 0.01), but lower cortical BMD scores (*p* < 0.01). Cortical BMD *Z*-scores were significantly lower in CKD stages 4–5 compared to healthy controls, and the duration of CKD also affected this (*p* < 0.05) [68]. A greater trabecular volumetric BMD was seen in younger participants, the reason for which is not entirely clear. It is postulated that this could be attributed to the anabolic effect of PTH, as this is not seen in other chronic conditions [99]. Lima et al. showed that in 21 patients on peritoneal dialysis, cortical BMD was decreased and this correlated with higher alkaline phosphatase and PTH levels. Trabecular BMD however was higher than in controls, likely due to higher PTH [100]. As described previously, Denburg et al. have shown that per one SD lower baseline cortical BMD *Z*-score, there was a 1.75-fold higher fracture risk (95% CI 1.15–2.67, *p* = 0.009) in children with CKD2-5D [43].

#### High-resolution pQCT

High-resolution pQCT (HRpQCT) uses a higher spatial resolution allowing for an even more detailed look at the trabecular bone microarchitecture and enabling measurement of trabecular numbers, thickness and separation [73]. Marques et al. have evaluated adult dialysis patients that underwent bone biopsy and HRpQCT [101] and showed that trabecular number, separation and thickness obtained from HRpQCT and from bone biopsy correlated and that patients with cortical porosity on bone histomorphometry presented lower cortical density at the distal radius. However, HRpQCT could not predict mineralisation abnormalities. Conversely, Pereira et al. showed that bone mineralisation might be assessed by HRpQCT in paediatric dialysis patients [102]. Ramalho et al. obtained DXA imaging of the spine for a trabecular bone score (TBS), and HRpQCT of the radius and tibia in 52 participants who had previously undergone bone biopsy. They showed that TBS reflected trabecular microarchitecture as assessed by bone biopsy, and TBS reflected cortical measures at the tibia as assessed by HRpQCT [103]. Finally, Preka et al. assessed vascular measures and HRpQCT in 32 children aged 10–17 years with CKD stages 2–5 and found an association of calcium and trabecular thickness with mean blood pressure [104].

#### Magnetic resonance imaging

Magnetic resonance imaging (MRI) and high-resolution MRI (HR-MRI) in particular have recently been explored as a more detailed way of non-invasively evaluating the bone structure, without the use of ionising radiation [105]. In fact, it is thought that it provides enough detailed information about the standard histomorphometry parameters and microarchitecture to be considered a ‘virtual biopsy’ by some [106]. It allows for detailed visualisation of both cortical and trabecular compartments and making it possible to see actual three-dimensional views of the architecture. The commonest sites used to obtain images are the distal radius, calcaneus and distal tibia [107, 108]. Adult studies have compared MRI to bone histomorphometry showing good correlation of studied indices across the two modalities [107, 109, 110]. There are no studies in children with CKD.

#### Bone quantitative ultrasound

Quantitative ultrasound (QUS) has also been used to assess bone as it is radiation-free, fast, inexpensive and readily available. The measurements are based on the attenuation of the ultrasound wave or speed of sound as it passes through the structure being examined. The penetration depth of the ultrasound waves means that only peripheral sites can be used: commonly, the calcaneus, radius, phalanges or tibia are used. In children, it is currently only used in research as an adjunct to other modalities [111]. Adamczyk et al. assessed seventy-six children with CKD and normal renal function who had received glucocorticoid treatment by lumbar and total body DXA as well as phalange QUS. The CKD group had the lowest total body DXA *Z*-scores and the QUS *Z*-scores compared to controls (*p* < 0.0001) [112]. Another study comparing lumbar spine DXA and radius and tibia QUS in 643 participants aged 5–20 years old (412 healthy, 117 with cystic fibrosis and 114 with a body mass index kg/m^2^ above 95th centile) showed a good correlation of the two modalities in healthy children. However, in children with cystic fibrosis or high BMI, there was a 6–31% disagreement of measurements. The authors concluded that the two modalities are not yet interchangeable in their use or interpretation [113]. The predictive value of QUS for fracture risk is yet to be explored.

## International guidelines on bone assessment in childhood CKD-MBD

Current management of CKD-MBD is based on keeping calcium, phosphate and PTH within an optimum range in order to maintain bone turnover, but without increasing the risk of ectopic calcification. This can be done by controlling plasma calcium and phosphate by dietary restrictions, phosphate binders, vitamin D, active vitamin D analogues and dialysis. These are discussed in the 2006 European Paediatric Dialysis Working Group (EPDWG) prevention and treatment of renal osteodystrophy guidelines, the 2013 National Institute for Health and Clinical Excellence (NICE) management of hyperphosphataemia guidelines [114] and the 2017 KDIGO CKD-MBD guidelines [3].

### On biomarkers and bone biopsy in children

The bone biomarkers studied so far have not been sensitive or specific enough to be translated into an accurate clinical tool to predict bone turnover and mineralisation states. The International Osteoporosis Foundation states that bone turnover markers may be useful in routine clinical practice to predict fracture risk and assess bone mineralisation, but they currently have significant drawbacks such as biological variability and inadequate evidence [10]. It recommends studying PINP as the main bone formation biomarker and CTX as the main bone resorption biomarker to aid standardisation [10].

Although PTH and BSAP levels have been associated with bone turnover, no biomarker is sufficiently robust to diagnose low, normal and high bone turnover in an individual patient. The 2017 KDIGO guidelines recommend using trends in PTH rather than absolute ‘target’ values when making decisions as to whether to start or stop treatments to lower PTH. When trends in PTH are inconsistent, a bone biopsy may be considered. The recommendation to perform a bone biopsy is based on the possibility it may affect treatment and the decision to administer antiresorptive therapy. Since this is rarely, if ever, done in children with CKD, the role of bone biopsies remains questionable.

### On bone imaging

The International Society for Clinical Densitometry (ISCD) 2007 and KDIGO 2009 guidelines discouraged routine DXA BMD testing in CKD3-5 since BMD does not predict the type of bone turnover. This is because hyperparathyroidism has generally catabolic effects on the cortical bone with a decrease in cortical volumetric BMD and cortical thickness whereas it exerts anabolic effects on trabecular bone. However, the 2017 KDIGO Guideline on treatment of CKD-MBD [3], based on four prospective cohort studies of DXA BMD and incident fractures in adults with CKD stages 3a to 5D [85, 86, 115, 116], demonstrated that DXA BMD predicted fractures across the spectrum from CKD stages 3a to 5D in adults. The guidelines therefore suggest that DXA BMD assessment should be considered if it will lead to additional treatments or therapy recommendations in adults [3, 85, 115].

For children with CKD, no studies have examined the association between DXA results and fractures, and so the KDIGO 2017 update does not provide specific recommendations for DXA use in children. DXA has been suggested as a potentially useful tool in assessing BMD in children, given that good correlations have been shown between DXA lumbar spine BMD and tibial pQCT measures [91], as well as the correlation between change in tibial cortical BMD and fracture risk [43]. The ISCD in their 2013 Official Positions document also suggests there may yet be a role for DXA in the assessment of BMD in children [76, 77]. Importantly, as children and adolescents with CKD frequently exhibit substantial growth failure, and DXA measures of areal BMD underestimate volumetric BMD in children with short stature [83], DXA results should be adjusted for bone size, consistent with the 2013 ISCD Paediatric Official Positions [76].

## Conclusion

Skeletal abnormalities are prevalent in children with CKD, affecting bone mineralisation from early CKD and manifesting as bone pains, deformities and fractures. The bone disease of childhood CKD can persist after transplantation and even manifest with osteoporosis and fractures in adulthood. Bone imaging, histology and biomarkers are variably used to assess bone disease in CKD, but there are few evidence-based studies to promote their use in routine clinical practice. Current clinical management, as advocated by the KDIGO 2017 guideline, is limited to the measurement and management of trends in serum calcium, phosphate, PTH, vitamin D and alkaline phosphatase, with DXA assessment of BMD if it is likely to influence clinical management.
